# A multi-model longitudinal assessment of ChatGPT performance on medical residency examinations

**DOI:** 10.3389/frai.2025.1614874

**Published:** 2025-08-22

**Authors:** Maria Eduarda Varela Cavalcanti Souto, Alexandre Chaves Fernandes, Ana Beatriz Santana Silva, Louise Helena de Freitas Ribeiro, Thales Allyrio Araújo de Medeiros Fernandes

**Affiliations:** ^1^Department of Biomedical Sciences, School of Health Sciences, State University of Rio Grande do Norte, Mossoró, Brazil; ^2^Institute of Mathematics and Computer Sciences, University of São Paulo, São Paulo, Brazil

**Keywords:** generative artificial intelligence, medical residency examinations, medical education, artificial intelligence, chain-of-thought reasoning, large language model

## Abstract

**Introduction:**

ChatGPT, a generative artificial intelligence, has potential applications in numerous fields, including medical education. This potential can be assessed through its performance on medical exams. Medical residency exams, critical for entering medical specialties, serve as a valuable benchmark.

**Materials and methods:**

This study aimed to assess the accuracy of ChatGPT-4 and GPT-4o in responding to 1,041 medical residency questions from Brazil, examining overall accuracy and performance across different medical areas, based on evaluations conducted in 2023 and 2024. The questions were classified into higher and lower cognitive levels according to Bloom’s taxonomy. Additionally, questions answered incorrectly by both models were tested using the recent GPT models that use chain-of-thought reasoning (e.g., o1-preview, o3, o4-mini-high) with evaluations carried out in both 2024 and 2025.

**Results:**

GPT-4 achieved 81.27% accuracy (95% CI: 78.89–83.64%), while GPT-4o reached 85.88% (95% CI: 83.76–88.00%), significantly outperforming GPT-4 (*p* < 0.05). Both models showed reduced accuracy on higher-order thinking questions. On questions that both models failed, GPT o1-preview achieved 53.26% accuracy (95% CI: 42.87–63.65%), GPT o3 47.83% (95% CI: 37.42–58.23%) and o4-mini-high 35.87% (95% CI: 25.88–45.86%), with all three models performing better on higher-order questions.

**Conclusion:**

Artificial intelligence could be a beneficial tool in medical education, enhancing residency exam preparation, helping to understand complex topics, and improving teaching strategies. However, careful use of artificial intelligence is essential due to ethical concerns and potential limitations in both educational and clinical practice.

## 1 Introduction

Artificial Intelligence (AI) has been progressively integrated across various fields due to AI models’ broad and promising applicability in the real world, spanning numerous work and research domains (Sarker, 2022; Haenlein and Kaplan, 2019). Among these AI models, ChatGPT, OpenAI’s natural language processing-based AI, stands out as an example of a model trained on a generalist dataset, gaining significant recognition since its launch in November 2022 (Liebrenz et al., 2023).

In the medical field, the rapid development of artificial intelligence tools is increasingly gaining importance, highlighting their promising potential in clinical practice and medical research. Among their potential functions are assistance in the collection, analysis, and interpretation of data, in scientific writing, in medical record documentation, and in supporting medical decision-making (Garg et al., 2023).

From the perspective of medical education, the new tools utilizing AI prompt reflections on how medical education should adapt to this new landscape. An example of this is the satisfactory performance in solving tests across various fields of knowledge, including a promising performance in the United States Medical Licensing Examination (USMLE) (Kung et al., 2023).

However, published studies have not thoroughly evaluated this technology’s performance in addressing medical residency exam questions in Brazil, and few have assessed whether chain-of-thought reasoning improves accuracy on complex clinical questions. Therefore, this study aims to assess the longitudinal effectiveness of different versions of ChatGPT in solving medical residency exams in Brazil and contribute new data to comprehensively evaluate AI performance.

## 2 Methods

### 2.1 Selection of questions

The performance of GPT-4 was tested on 1,041 medical residency exam questions from the years 2022 (601 questions) and 2023 (440 questions), which were publicly available on the websites of nationally renowned institutions. Questions that included non-textual elements, such as images, tables, or charts, were not considered due to the evaluated AI’s inability to process such data types at the start of the analyses. Questions that were nullified by the original examining board were excluded. This study did not require approval from an institutional review board (IRB) or ethics committee because it used only publicly available data and did not involve human subjects. The dataset can be found in the Data Availability Statement section.

### 2.2 AI model versions and evaluation timeline

The study involved a longitudinal evaluation of several model versions. For each assessment, the specific model version tested was the one available on the official ChatGPT web interface during the corresponding period. The analysis began with GPT-4 (September 6 to September 30, 2023), which had been pre-trained with information available up to September 2021 (OpenAI, 2024); followed by GPT-4o (July 29 to September 10, 2024). All questions that both models failed were subsequently retested with GPT o1-preview (September 18, 2024), released on September 12, 2024, and again in April 2025 using the newer GPT-o3 and o4-mini-high models, both released that same month.

### 2.3 Classification and organization of questions

The questions were selected and organized into a spreadsheet, where they were classified into five major areas (Internal Medicine, Surgery, Preventive Medicine, Gynecology and Obstetrics, and Pediatrics), as well as general information described for each question in their columns: exam institution, question text and answer options, official answer key, and a column for the AI responses. This classification into five major areas reflects the standard structure of Brazilian medical residency exams, where questions are typically organized into blocks covering these areas in roughly equal proportions. When the area was not explicitly specified in the exam materials, classification was confirmed by a physician with expertise in medical education based on question content.

To determine if the complexity of the statements in incorrect questions was greater than that of the correct ones, a health education researcher categorized all questions into groups representing higher-order and lower-order thinking, reflecting the hierarchy from less to greater cognitive processing, as defined by Bloom’s Taxonomy (Adams, 2015; Tuma and Nassar, 2021). To ensure the human evaluator’s complexity analysis was not influenced, the spreadsheet was revised to remove any columns revealing the institution of origin, the medical area, or the AI’s responses. Consequently, the document had only a numeric identification code for each question and the column containing the respective statement and options.

### 2.4 Interactions with ChatGPT

ChatGPT was given instructions on the task of answering multiple-choice questions. To this end, a key command was crafted for problem-solving through prompts, a programming method that can customize results and interactions with ChatGPT based on specific patterns to ensure adherence to certain rules (White et al., 2023). Each question was entered into ChatGPT as a single message. All questions were manually entered into the large language models via the official ChatGPT web interface. The OpenAI API was not used for this study to ensure the testing environment reflected typical user interaction. Once the responses were gathered, the alternatives identified as correct by the AI were recorded on the constructed spreadsheet.

### 2.5 Statistical analysis

The data were analyzed using the open-source statistical software Jamovi version 2.3.28. Overall accuracy was determined as the ratio of correct answers to the total number of questions. Accuracy across each of the five medical areas was also examined. The 95% confidence intervals (CI) for accuracies were computed for overall accuracy and for each area. The statistical significance of the accuracy comparison between models and between higher-order and lower-order Bloom’s taxonomy question groups was evaluated using the two-proportion Z-test.

## 3 Results

### 3.1 Accuracy

**Figure 1:**
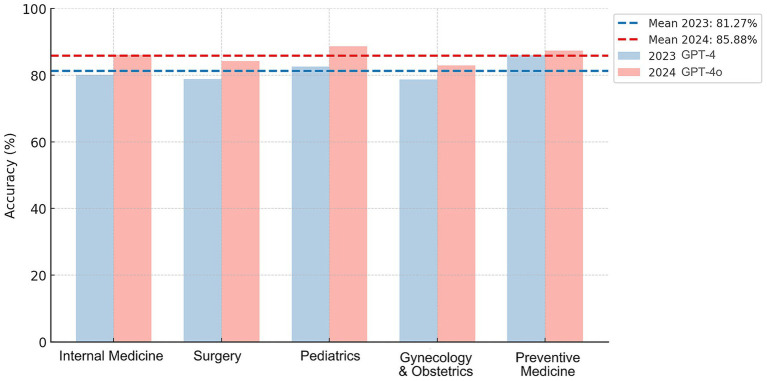
Average accuracy across five medical fields. The bar chart displays the average accuracy for the five major areas. The bars indicate the percentage mean of correct answers in each category.

Out of the 1,041 questions analyzed, 846 correct answers were obtained from the GPT-4 model, representing an overall accuracy of 81.27% (95% CI: 78.89–83.64%). The area with the highest accuracy was preventive medicine, while the lowest was gynecology and obstetrics (Figure 1; Table 1). The GPT-4o achieved 894 correct answers, representing an overall accuracy of 85.88% (95% CI: 83.76–88.00%). The area with the highest accuracy was pediatrics, while the lowest accuracy was in gynecology and obstetrics (Figure 1; Table 1). The comparison of the statistical significance of GPT-4o over GPT-4 resulted in *p* < 0.05.

**Table 1:** Number of questions and accuracy percentages for different medical fields, along with the 95% Confidence Interval (CI) for each medical field, based on the performance of the GPT-4 model in 2023 and GPT-4o model in 2024.

Medical field	Number of questions	Accuracy GPT-4 (2023)	95% CI	Accuracy GPT-4o (2024)	95% CI
Surgery	203	78.82%	(73.15–84.49)	84.24%	(79.18–89.29)
Internal medicine	201	80.10%	(74.53–85.67)	86.07%	(81.24–90.90)
Gynecology and obstetrics	211	78.67%	(73.10–84.25)	82.94%	(77.82–88.06)
Preventive medicine	214	85.98%	(81.29–90.67)	87.38%	(82.90–91.87)
Pediatrics	212	82.55%	(77.40–87.70)	88.68%	(84.38–92.98)

### 3.2 Complexity level of questions by bloom’s taxonomy

Within the context of Bloom’s taxonomy, 541 questions were classified as higher-order, while 500 were considered lower-order. For the lower-order question group, GPT-4 showed an accuracy of 83.40% (95% CI: 80.13–86.67%), while GPT-4o showed 87.20% (95% CI: 84.26–90.14%). In the higher-order group, GPT-4 demonstrated an accuracy of 79.30% (95% CI: 75.87–82.72%), and GPT-4o achieved 84.66% (95% CI: 81.61–87.70%). The detailed data for each area in the higher-order and lower-order groups can be observed in Tables 2, 3.

**Table 2:** Number and accuracy of lower-order questions as defined by Bloom’s taxonomy for different medical fields, along with the 95% confidence interval (CI) for each medical field, based on the performance of the GPT-4 model in 2023 and GPT-4o model in 2024.

Medical field	Number of questions	Accuracy GPT-4 (2023)	95% CI	Accuracy GPT-4o (2024)	95% CI
Surgery	117	81.20%	(74.01–88.38)	82.91%	(75.98–89.83)
Internal medicine	60	86.67%	(77.81–95.52)	93.33%	(86.84–99.83)
Gynecology and obstetrics	91	80.22%	(71.88–88.56)	84.62%	(77.06–92.17)
Preventive medicine	138	87.68%	(82.13–93.23)	89.13%	(83.87–94.39)
Pediatrics	94	80.85%	(72.75–88.95)	88.30%	(81.68–94.92)

**Table 3:** Number and accuracy of higher-order questions as defined by Bloom’s taxonomy for different medical fields, along with the 95% confidence interval (CI) for each medical field, based on the performance of the GPT-4 model in 2023 and GPT-4o model in 2024.

Medical field	Number of questions	Accuracy GPT-4 (2023)	95% CI	Accuracy GPT-4o (2024)	95% CI
Surgery	86	75.58%	(66.32–84.85)	86.05%	(78.57–93.52)
Internal medicine	141	77.30%	(70.31–84.30)	82.98%	(76.70–89.26)
Gynecology and obstetrics	120	77.50%	(69.92–85.08)	81.67%	(74.64–88.69)
Preventive medicine	76	82.89%	(74.23–91.56)	84.21%	(75.82–92.60)
Pediatrics	118	83.90%	(77.17–90.63)	88.98%	(83.25–94.72)

The comparison between the lower-order and higher-order groups using the Two-proportion Z-Test resulted in a *p*-value of 0.09 in the GPT-4 model and 0.239 in the GPT-4o model.

### 3.3 Accuracy of chain-of-thought reasoning models on incorrect responses from both GPT-4 and GPT-4o

All 92 questions that both previous models (GPT-4 2023 and GPT-4o 2024) answered incorrectly were selected and used to evaluate three newer versions: GPT o1-preview (September 2024), GPT o3 (April 2025), and GPT o4-mini-high (April 2025). The overall accuracy was 53.26% (95% CI: 42.87–63.65%) for o1-preview, 47.83% (37.42–58.23%) for GPT o3, and 35.87% (25.88–45.86%) for o4-mini-high. All models performed better on higher-order questions than on lower-order ones, with statistically significant differences for o1-preview (63.46% on higher-order and 40.00% on lower-order, *p* = 0.025) and GPT o3 (59.62% on higher-order and 32.50% on lower-order, *p* = 0.010), though not for o4-mini-high (42.31% on higher-order and 27.50% on lower-order, *p* = 0.142).

## 4 Discussion

The findings indicate a promising overall performance of the tool, achieving a pass rate that would secure approval in the first phase of selection across various direct-access medical residency specialties in Brazil. Additionally, it is observed that AI models are improving their accuracy as new versions are released.

Understanding the structure of the Brazilian medical residency examination system is important to interpret these results. The system consists of independent selection processes conducted by each institution. The exams are administered exclusively in Portuguese and are designed for general practitioners seeking admission into medical residency programs. They assess candidates across five core medical areas: Internal Medicine, Surgery, Pediatrics, Gynecology and Obstetrics, and Preventive Medicine. Admission is highly competitive and rank-based, with candidates admitted according to their relative performance to fill a limited number of available positions. Consequently, there is no universal passing score.

This performance aligns with other international studies that have tested the performance of ChatGPT-4 in different medical contexts (Zong et al., 2024; Huang et al., 2023; Goodings et al., 2024). For instance, regarding the UK’s dermatology specialist exam performance, ChatGPT 4 achieved an average score of 90.5% (Passby et al., 2024). In the context of neurosurgery questions, it performed at 82.6% (Ali et al., 2023). In Brazil, a study testing its performance in the Dermatology board exam achieved a score of 79.17% in the 2022 exam (Jabour et al., 2023). These outcomes suggest a promising performance across various settings and significant educational potential. However, these results should be interpreted cautiously in clinical contexts, as the ability to perform well in written tests is just one of many medical skills, and should not be seen as a substitute for the entire clinical process, which also involves cognitive clinical reasoning skills and practical abilities, for example.

The field with the lowest performance was gynecology and obstetrics, suggesting that the database might have been trained on less information from this area. Another possibility is the more significant global heterogeneity of knowledge in this field, considering that performance was tested on a nationality-specific examination. A significant observation is that the performance heterogeneity across fields may indicate that the tool, at least in its current form, could serve as a more reliable educational aid in certain areas than in others. Furthermore, this raises the hypothesis that in the future, there could be increasingly personalized models, specifically trained for certain fields, to achieve more significant and specific accuracy (Martínez-García and Hernández-Lemus, 2022), aligning more closely with the concept of precision medicine.

GPT-4 and GPT-4o demonstrated better performance on lower-order questions as classified by Bloom’s taxonomy. This outcome aligns with expectations, where questions requiring higher levels of interpretation and analysis are possibly more challenging to address than those that merely involve applying memorized information, as is the case with lower-order questions. Our findings are consistent with another study that evaluated ChatGPT’s performance on the American Board of Radiology certification exam, where higher-order questions had a lower average of correct answers (Bhayana et al., 2023). However, in the results of the present study, the models that use chain-of-thought reasoning (GPT o1-preview, o3, and o4-mini-high), demonstrated superior performance in the higher-order question group. This finding may be attributed to this model’s use of the chain-of-thought mechanism, which enhances the ability of large language models to solve more complex problems (Wei et al., 2022).

In this promising context, understanding the potential of AI use, such as ChatGPT, in educational and evaluative settings can contribute to advancing machine learning in the medical field. Furthermore, it can provide a foundation for AI applications as an auxiliary tool in preparing candidates for residency exams or formulating assessment questions (Shoja et al., 2023).

It is essential to emphasize the exponential growth of knowledge published in healthcare (Kundu, 2021). Given this, to stay up-to-date with primary care literature, for example, a medical student would need to study over 29 h every weekday (Kundu, 2021). This scenario highlights the need for strategies and technologies to help manage this vast amount of information, enabling students and medical professionals to fully engage with the high volume of knowledge production through these auxiliary technological systems. In this context, medical education should focus on developing skills and critical thinking, reducing the excessive emphasis on memorization.

In this context, within the landscape of AI and other tools that enhance the capabilities of managing medical knowledge, the importance of competency-based medical learning and the development of soft skills becomes even more critical. Medical education must extend beyond the teaching of biomedical and clinical sciences, also fostering skills such as communication, leadership, teamwork, flexibility, creativity, proactivity, and empathy, which are essential in the medical work environment (Wartman and Combs, 2018; Lee et al., 2021).

Access to and inclusion in these technologies must also be addressed, as the infrastructure and technology required to enable their use come with significant costs, potentially limiting their application in economically disadvantaged countries and regions (Guidance for generative AI in education and research, 2023). In this context, an important concept is Artificial Intelligence in Education (AIED) unplugged, which refers to harnessing the potential of AI in education without relying on extensive local infrastructure, instead leveraging the existing technological resources available in these areas (Isotani et al., 2023).

Another essential aspect to consider is the ethical issues involved in the application of this AI in medical education, as its use could violate copyright laws, lead to medico-legal implications due to misuse, and present dilemmas regarding the accountability for the material produced (Dave et al., 2023). Additionally, it is important that AI models undergo training aligned with human values and principles, and that they are properly supervised (Yu et al., 2024).

This study has several limitations. There was no direct benchmarking against human performance. Model evaluations were conducted at different time points as new versions became available. The analysis was also restricted to a single language context. Future research should address these methodological constraints and, importantly, move beyond exam accuracy to rigorously evaluate the real-world educational impact of such technologies. Further studies are needed to clarify the role of AI in supporting the acquisition of essential skills and competencies in medical education. Therefore, generative AI, represented in this study by ChatGPT, is a promising technology across various fields, including medical residency contexts. AI should evolve toward more personalized models with high accuracy in specific areas. However, the potential applications and associated ethical dilemmas must be carefully evaluated (Weidener and Fischer, 2023; Weidener and Fischer, 2024). This progress further emphasizes the importance of medical education, focusing on developing competencies, skills, and critical thinking.

## Data Availability

The data analyzed in this study is subject to the following licenses/restrictions: The original exam questions are publicly accessible through official Brazilian residency examination websites. The model-generated answers to these questions can be requested directly from the first author via email or https://github.com/mariaevcsouto/chatgpt-multimodel-medexam-benchmark. Requests to access these datasets should be directed to mariaevcsouto@gmail.com.
