# Supplementary figures and images for: A multi-model longitudinal assessment of ChatGPT performance on medical residency examinations
Source: Front Artif Intell. 2025 Aug 22;8:1614874. doi: 10.3389/frai.2025.1614874 (PMC12411524; doi:10.3389/frai.2025.1614874)

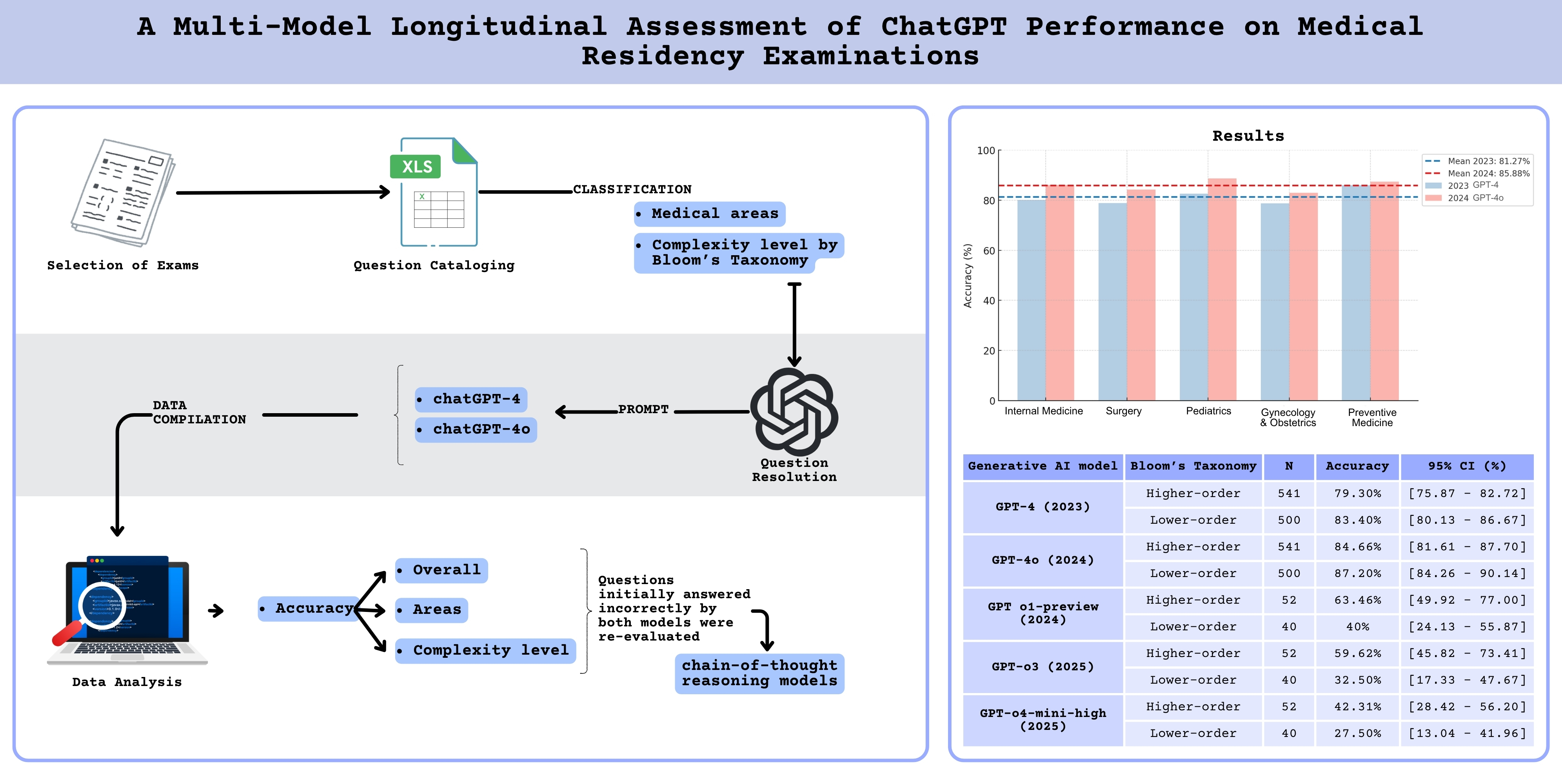

Supplement: Supplementary file 1 [file Image_1.jpeg]
